# Genes implicated in thiopurine-induced toxicity: Comparing TPMT enzyme activity with clinical phenotype and exome data in a paediatric IBD cohort

**DOI:** 10.1038/srep34658

**Published:** 2016-10-05

**Authors:** Tracy Coelho, Gaia Andreoletti, James J. Ashton, Akshay Batra, Nadeem Ahmad Afzal, Yifang Gao, Anthony P. Williams, Robert M. Beattie, Sarah Ennis

**Affiliations:** 1 Human Genetics and Genomic medicine, University of Southampton, Southampton, UK; 2 Department of Paediatric Gastroenterology, University Hospital Southampton, Southampton, UK; 3 Cancer Sciences Division, Faculty of Medicine, University Hospital Southampton, Southampton, UK

## Abstract

The aim of our study was to assess the utility of next generation sequencing (NGS) for predicting toxicity and clinical response to thiopurine drugs in paediatric patients with inflammatory bowel disease. Exome data for 100 patients were assessed against biochemically measured TPMT enzyme activity, clinical response and adverse effects. The *TPMT* gene and a panel of 15 other genes implicated in thiopurine toxicity were analysed using a gene based statistical test (SKAT-O test). Nine patients out of 100 (Crohn’s disease- 67, ulcerative colitis- 23 and IBDU-10) had known *TPMT* mutations associated with deficient enzyme activity. A novel and a highly pathogenic *TPMT* variant not detectable through standard genotyping, was identified through NGS in an individual intolerant to thiopurines. Of the 14 patients intolerant to thiopurines, NGS identified deleterious *TPMT* variants in 5 individuals whereas the biochemical test identified 8 individuals as intolerant (sensitivity 35.7% and 57.14%; specificity 93.75% and 50% respectively). SKAT-O test identified a significant association between *MOCOS* gene and TPMT activity (p = 0.0015), not previously reported. Although NGS has the ability to detect rare or novel variants not otherwise identified through standard genotyping, it demonstrates no clear advantage over the biochemical test in predicting toxicity in our modest cohort.

Thiopurine drugs, which include azathioprine and 6-mercaptopurine (6-MP) have been effectively used in inflammatory bowel disease for more than 30 years. They are also widely used in the treatment of patients with neoplastic conditions, post-organ transplantation and a wide range of autoimmune and inflammatory conditions. However concerns over toxicity and adverse reactions frequently result in discontinuation of treatment and a switch to alternative therapies^1^,^2^. Known adverse reactions include bone marrow suppression, severe gastric intolerance, pancreatitis, hepatotoxicity, skin reactions, susceptibility to infections, risk of malignancy and flu-like symptoms^2^,^3^.

Thiopurine S-methyl transferase (TPMT) is a key enzyme involved in the metabolism of thiopurine drugs and functions by catalysing the S-methylation of aromatic and heterocyclic sulfhydryl groups^4^. TPMT is encoded by a gene located on chromosome 6p22, consisting of 10 exons, encoding one protein domain^5^. Although the precise mode of action of thiopurines is still unclear, the most important mechanism is thought to be the incorporation of 6-TGNs (thioguanine nucleotides) into the cell DNA, resulting in an impaired DNA synthesis and cell death^2^,^6^ (Fig. 1). The *TPMT* gene is known to exhibit genetic heterogeneity resulting in wide inter-individual differences, both in terms of clinical efficacy and toxicity profiles based on the enzyme activity^2^,^7^. Approximately 4–11% of individuals of Caucasian origin are heterozygous for a mutant *TPMT* allele with intermediate enzyme activity, whereas approximately 1 in 300 individuals are homozygous or compound heterozygous with a consequent low or absent *TPMT* activity^3^,^7^. Current clinical guidelines recommend determining *TPMT* status in a given patient before commencement of thiopurine therapy; this is achieved by measuring *TPMT* enzyme activity in the circulating red blood cells *or* through genotyping known *TPMT* variants associated with enzyme deficiency^2^,^8^,^9^.

The rationale for assessing the TPMT status before commencement of thiopurine therapy is to minimise the risk of adverse effects whilst aiming for an optimal clinical response. Thiopurines are best avoided in individuals who are deficient or have extremely low TPMT activity and administered at a reduced dose if TPMT activity is intermediate or low normal^2^,^9^,^10^.

Although *TPMT* is the most crucial pharmaco-gene involved in the metabolism of thiopurines, previous studies have highlighted the role of other genes, whose products substantially alter drug metabolism and consequently impact clinical efficacy or toxicity^11^,^12^,^13^,^14^,^15^,^16^,^17^,^18^. It is plausible that some proportion of adverse effects observed whilst on treatment in the context of a normal *TPMT* status (genotype or phenotype), could be explained by variation in genes encoding the other enzymes involved in thiopurine metabolism.

In this study, we identify genes known to be involved in thiopurine metabolism and determine all coding mutations in these genes in a cohort of 100 children with IBD.

We assess the joint effect of rare and common variants within the *TPMT* gene and other genes implicated in thiopurine toxicity on TPMT enzyme activity through the application of a gene based statistical test (SKAT-O). The test was also conducted to investigate the association of these variants with thiopurine tolerance and clinical response.

## Materials and Methods

### Study Population

Patients were identified through the paediatric gastroenterology service database based at the University Hospital Southampton (UHS), recruited from outpatient clinics and followed through their treatment. All children were diagnosed using the Porto diagnostic criteria^19^ and treated according to British Society of Paediatric Gastroenterology, Hepatology and Nutrition (BSPGHAN) published guidelines^9^. Data for one hundred paediatric patients with TPMT phenotype defined as red blood cell enzyme activity and concurrent exome data were analysed.

### Ethical Approval

The study was ethically approved by Southampton and South West Hampshire Research Ethics Committee (09/H0504/125). Informed consent was obtained from all participants before recruitment to the study. All methods were carried out in accordance with the approved and published guidelines.

### TPMT Phenotype Determination

TPMT enzyme activity was measured using standard high performance liquid chromatographic technique^20^. TPMT enzyme activity level groups were defined as previously described (Fig. 2)^20^,^21^.

### Use of Thiopurines and Monitoring for Adverse Effects

British Society of Paediatric Gastroenterology, Hepatology and Nutrition (BSPGHAN) recommend initiation of treatment with thiopurines for maintenance of remission in individuals who relapse in less than 6 months, have 2 or more relapses per year following initial successful therapy and in all steroid-dependent patients. Practice with regards to initiation of treatment varies among clinicians, but is usually commenced and monitored as per the BSPGHAN guidelines^9^,^19^, with regular blood tests to monitor adverse effects such as bone marrow suppression, pancreatitis, hepatotoxicity and patients are clinically followed up to assess progress through treatment. For this study, bone marrow suppression was defined as leucopoenia (WBC < 3000 mm^−3^) and/or thrombocytopenia (platelets < 100,000 mm^−3^); liver toxicity was alanine transaminase (ALT), gamma-glutamyl transpeptidase (GGT) or alkaline phosphatase more than twice their normal levels; acute pancreatitis was defined as significant abdominal pain within 3 months of starting thiopurines, accompanied by a serum amylase or lipase level of greater than twice their normal levels as per our local laboratory values^22^.

### Evaluation of response to therapy

Assessment of clinical poor response or non-response was based on one of the following: (1) Inability to achieve clinical improvement as assessed by global clinical assessment after at least 6 months of thiopurine therapy; (2) Corticosteroid dependence after at least 3 months of thiopurine therapy; (3) Relapse within 6 months of therapy; (4) Use of biologics within 6 months of therapy with thiopurines and; (5) Disease progression needing surgery within 6 months of thiopurine therapy commencement.

Intolerance to treatment was based on all of the following: (1) Occurrence of adverse side effects (including bone marrow suppression, severe gastric intolerance, pancreatitis, hepatotoxicity, skin reactions, flu-like symptoms); (2) Adverse effects unexplained by disease course or other concomitant co-morbidities; and (3) Partial or complete resolution of the observed adverse effects following discontinuation of therapy.

### Genes Implicated in Thiopurine Toxicity

A systematic search was conducted through ovidsp using MEDLINE and EMBASE from inception to the end of March 2015. Only studies in humans describing *TPMT* genetic variants and other genes involved in thiopurine metabolism and toxicity were included. We applied the *TPMT* nomenclature committee website (http://www.imh.liu.se/tpmtalleles) that outlines all reported *TPMT* variants^23^, to cross-reference against variants identified in this study.

## Whole Exome Sequencing and Data Analysis

### DNA Extraction

Genomic DNA was extracted from peripheral venous blood samples collected in EDTA, using the salting out method as previously described^24^,^25^.

### Exome Sequencing

Whole-exome capture was performed using Agilent SureSelect Human all Exon 51 Mb (versions 4 and 5) capture kit as previously described^25^. A bespoke script was used to assign individual variants as “novel” if they were not previously reported in the dbSNP137 databases^26^, 1000 Genomes Project (1 KG)^27^, the Exome Variant Server (EVS) of European Americans of the NHLI-ESP project with 6500 exomes [http://evs.gs.washington.edu/EVS/], in 46 unrelated human subjects sequenced by Complete Genomics^28^ or in the Southampton database of reference exomes.

### Burden of Mutation Testing

The burden of genetic variations within the genes was conducted using a gene-based statistical test (the sequence kernel association optimal unified test- SKAT-O)^29^. To conduct the test, a group file of non-synonymous, synonymous, splicing, frameshifts and non-frameshift, stop gain and stop loss mutations was created for each of the genes analysed. SKAT-O was executed with the small sample adjustment, by applying MAF threshold of 0.05 to define rare variations within the whole cohort, and using default weights. The EPACTS software package^30^ was use to perform this test. The test was conducted to assess the impact of variations on TPMT enzyme activity, drug tolerance and clinical response.

## Results

### Clinical Data

Of the 100 patients, 78 initiated thiopurines as part of their clinical management while 22 maintained remission without recourse to this therapy. The median duration of follow up for the 78 patients commenced on thiopurines was 46 months (7–156) from diagnosis and 43 months (6–119) from starting therapy with thiopurines (Table 1).

### Genes identified in TMPT metabolism and toxicity

**Figure 1:**
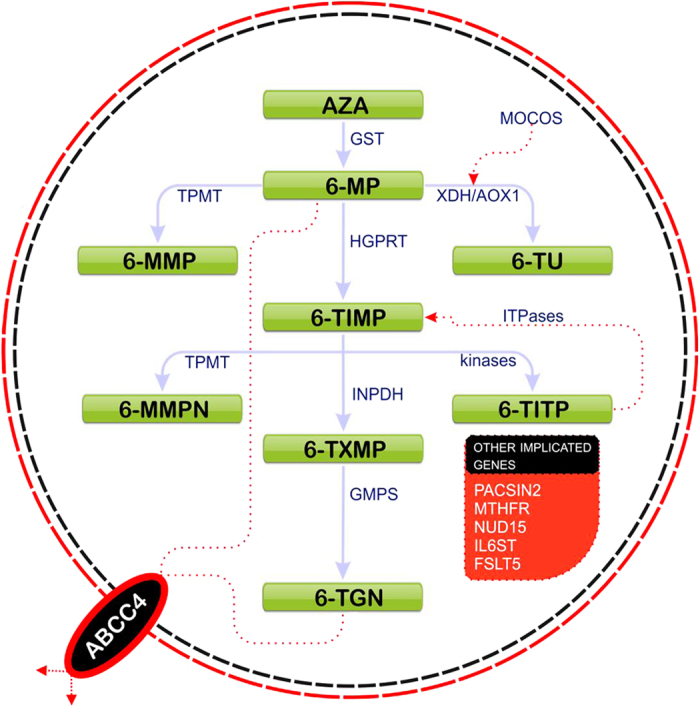
Schematic diagram showing thiopurine drug metabolism and genes implicated in thiopurine-induced toxicity. There are three main catabolic pathways for thiopurine drugs, following conversion of AZA to 6-MP: (1) Phosphorylation to 6-thioguanines (6-TGN) which are active metabolites; 6-MP is first converted by hypoxanthine guanine phosphoribosyl-transferase (HGPRT) to 6-thioinosine monophosphate (6-TIMP), which is then phophorylated to 6-TGN with inosine 5-monophosphate dehydrogenase (IMPDH1 and IMPDH2) and guanosine monophosphate synthetase (GMPS) (2) Methylation by of 6-MP by TPMT to form 6-methyl-MP (6-MMP) which is an inactive metabolite and not a substrate for IMPDH) (3) Catabolism of 6-MP to 6-thiouracil (6-TU) via xanthine dehydrogenase (XDH, synonym- Xanthine oxidase) or aldehyde oxidase 1 (AOX1). TPMT competes with IMPDH for their common substrate 6-TIMP to form 6- methylmercaptopurine nucleotides (6-MMPN). 6-TIMP can be phosphorylated by kinases to 6-thioinosine triphosphate (6-TITP), which can get dephosphorylated by inosine triphosphatase (ITPase) to form 6-TIMP again. Although the precise mode of action of thiopurines is still unclear, the most important mechanism is thought to be the incorporation of 6-TGNs into the cell DNA, resulting in an impaired DNA synthesis and cell death^2^,^6^. **Abbreviations:** ABCC4- ATP-binding cassette, sub-family C (CFTR/MRP), member 4; AZA- azathioprine; FSLT5- Follistatin-Like 5; GST- glutathione s-transferase; HGPRT- hypoxanthine phosphoribosyltransferase;IL6ST- Interleukin 6 signal transducer; 6-MP- 6- mercaptopurine; 6-MMPN- 6- methyl mercaptopurine nucleotides; MOCOS- Molybdenum cofactor sulfurase; MTHFR- Methyl-enetetrahydrofolate reductase ;NUDT15- Nudix (nucleoside diphosphate linked moiety X)-type motif 15; PACSIN2- Protein kinase C and casein kinase substrate in neurons 2; 6-TXMP- 6-thioxanthosine monophosphate)

A systematic search of genes implicated in TPMT metabolism and toxicity identified 15 genes with robust evidence from a review of over 3,000 articles (Fig. 1 and Supplementary Table S1) for which we could ascertain variation from exome data. Variation in *TMPT* and these genes was included in downstream analysis of: (1) biochemically assessed TMPT activity; (2) tolerance to thiopurines and; (3) response to treatment.

### Biochemically measured TPMT activity in red blood cells

**Figure 2:**
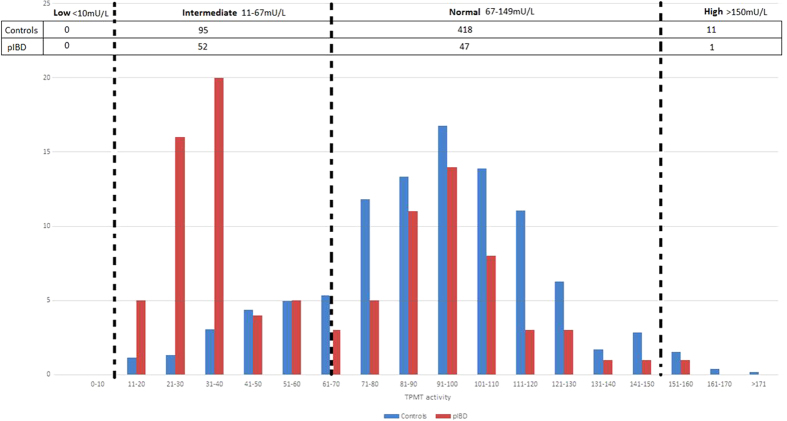
TPMT phenotype frequency distribution for the Wessex paediatric population and the research cohort. Figure shows the TPMT phenotype frequency distribution for 524 paediatric patients, in the Wessex region (524 patients ≤ 18 years, between Dec 2010–April 2015, includes patients with/without IBD) and the TPMT phenotype distribution in our research cohort. Within our cohort we observe a statistically significant difference between the sub-category with TPMT values between 21–40 units (p = 0.001) compared to the Wessex paediatric population. This difference is not observed between the sub-categories with TPMT values between 41 to161 (p = 0.90)

The TPMT enzyme activity was unexpectedly bimodal in distribution across the 100 patients examined in this study. We compared the distribution in our pIBD cohort against the same measure for an independent control group of individuals with various clinical diagnoses, aged ≤18 years from the Wessex region for whom TPMT activity was assessed by the same laboratory over the same period and observed a statistically significant distribution between the groups (P = 7.69 × 10^−12^) (Fig. 2). While approximately half of our cohort samples had biochemical activity levels within the normal range and half in the intermediate range, almost 80% of control samples had biochemical activity levels falling within the normal range. We believe this may reflect an ascertainment bias in our study cohort due to the fact that exome sequencing was preferentially conducted on children with most severe disease at earliest onset. This will have enriched for children with poor response to first-line treatments.

### *TPMT* gene variants - their correlation with biochemically measured TPMT enzyme activity, thiopurine tolerance and response

We identified five *TPMT* variants across our cohort (Table 2). These included two non-synonymous variants (A154T and Y240C) previously known to impact TMPT function that were found to co-segregate in 9 individuals. The mean biochemical measurement of TMPT activity for this group was 36 mU/L (range 16–56), which is significantly different (p = 0.003) from that found in the 91 individuals without these mutations (mean = 68.1 mU/L). Although exome data are insufficient to resolve haplotypes, we postulate these two variants occur on a single haplotype in all nine individuals, consistent with the complex *TPMT* allele referred to as TPMT*3A, previously described as the most prevalent *TPMT* deficiency allele in Caucasians^31^. Of these 9 patients, 8 were commenced on thiopurine treatment and four were later identified as intolerant to this therapy. All four patients that tolerated thiopurine drugs showed therapeutic response.

One novel non-synonymous variant (p.A73V) was identified in a female patient with Crohn’s disease and a biochemical activity level of 55 mU/L. *In silico* tools indicate this variant is strongly conserved (Phylop = 0.99) and likely to have a deleterious impact on enzyme function (GERP = 4.98). The patient was initiated on thiopurine treatment at a reduced dose (1 mg/kg) but developed severe persistent nausea and treatment was discontinued. This variant has since been catalogued as a functionally significant allele by the *TPMT* nomenclature committee (http://www.imh.liu.se/tpmtalleles) with a unique allele number TPMT*39.

One UC patient harboured a rare intronic variant (in heterozygote form) proximal to the exon 7 splice junction (c.420-4G > A), this patient had a biochemical activity level of 32 mU/L, was administered thiopurine treatment which was tolerated and subsequently demonstrated therapeutic response. The MaxEnt score (0.95) for this splice junction variant predicts minimal impact on splicing within the gene. While this is positively consistent with drug tolerance, it does not explain the relatively low biochemical activity level observed in this patient.

Finally, we observed a common synonymous variant (I158I) at high frequency (37 heterozygotes and 57 homozygotes). Mean biochemical activity level is 67.1 mU/L (32–99) and 66 mU/L (152-145) across the subgroup of individuals carrying the variant in heterozygous and homozygous state respectively. The very common frequency of this variant and the fact it does not alter amino acid composition is consistent with silent variation having no impact on function.

### Burden of mutation testing within *TPMT and* additional genes implicated in thiopurine toxicity

We interrogated exome data across fifteen additional genes implicated in thiopurine metabolism and toxicity. We observed mutations across 11 genes (no variation in *HPRT1*, *GSTM1*, *FSLT5* and *MTHFR*).

SKAT-O test was applied to investigate the joint effect of rare, low frequency and common variations within these genes on TPMT enzyme activity, thiopurine tolerance and response.

Significant evidence for association was observed within *MOCOS* (p = 0.0015) and *TPMT* (p = 0.0017) gene with TPMT biochemical activity levels. The test also detected a nominal association between *GMPS* and drug tolerance (p = 0.0212) as well as variations within *IL6ST* (p = 0.0084) and *ABBC4* (p = 0.0452) and drug response (Table 3, Supplementary Tables S2 and S3).

### Individual tolerance and intolerance

Thiopurine drugs were discontinued in 14 individuals due to adverse effects. Eight of these patients had TPMT enzyme activity in the intermediate category and the rest had TPMT activity in the normal range.

Four of the eight individuals with TPMT enzyme activity in the intermediate range had known *TPMT* mutations associated with deficient enzyme activity and one individual had a novel *TPMT* mutation described in the previous section. In the rest of the nine individuals, there was enrichment for deleterious variants within the *MOCOS* gene and the *AOX1* gene (Table 4).

Prediction of thiopurine toxicity showed a specificity of 93.75% through detection of *TPMT* risk variants compared to 50% for the biochemical test. Both tests had a low sensitivity of 37% and 57% respectively for predicting toxicity (Table 5). However, all the five patients with deleterious variants within the *TPMT* gene detected through NGS were also identified as potentially intolerant through the biochemical test. Although the biochemical test identified a higher number of individuals intolerant to thiopurines, the difference between the two approaches in predicting toxicity was not statistically significant (P value 0.45, Fisher’s exact test). The clinical data of individuals intolerant to thiopurines, is shown in Table 6 and of the entire cohort in Supplementary Table S4.

## Discussion

It is well established that in a small percentage of patients, *TPMT* genotyping alone or in combination with TPMT phenotype is insufficient to predict tolerance to thiopurine drugs^2^. Several *TPMT* variants associated with deficient enzyme activity have been described^23^, however an appreciable subset of patients with intermediate to low activity do not harbour known risk alleles^32^. Our study identified nine individuals with the known *TPMT* mutations (9% compared to 11% reported in previous studies)^31^. All nine individuals had TPMT enzyme activity levels in the intermediate range in line with expectations; mean TPMT value across this group was significantly different compared to individuals without these mutations (36.6 mU/L compared to 68.1 mU/L, p = 0.003). However 43% of individuals with TPMT activity in the intermediate range did not have the known *TPMT* mutations. Our results indicate that although prediction of thiopurine toxicity through NGS has a higher specificity compared to the biochemical test, the sensitivity of both methods is clinically suboptimal (35.7% and 57.14% respectively). Among the 14 individuals intolerant to thiopurines, all the 5 patients who harboured deleterious *TPMT* variants would also have been predicted as potentially intolerant to thiopurines through the biochemical test. Furthermore, all the nine individuals with deleterious variants within the *TPMT* gene across the cohort of 100 patients would also have been identified as potentially intolerant as all these nine individuals had TPMT enzyme activity levels within the intermediate range. Hence based on first principles, NGS did not have a clear advantage over the biochemical test in predicting thiopurine toxicity.

We identified a highly pathogenic novel variant in *TPMT* in an individual who had TPMT enzyme activity level of 55 mU/L, but developed severe gastrointestinal toxicity despite a reduced dose. This suggests that thiopurine toxicity can develop in individuals harbouring rare or yet unknown variants, not detected through standard genotyping. As a widely used practical approach, *TPMT* genotyping of known variants is considered only when biochemical tests suggest a deficiency or if a patient has recently been transfused. However, a normal genotype for known variants cannot exclude the possibility of rare variation causing TPMT deficiency and development of adverse effects^2^. Exome sequencing therefore can be a powerful tool in identifying individuals at risk of toxicity who could have been missed through standard *TPMT* genotyping.

The SKAT-O test identified a significant association between variations within the *MOCOS* gene and TPMT enzyme activity (p = 0.0015). Molybdenum cofactor sulfurase (*MOCOS*) is a protein-coding gene located on 18q12, which sulfurates the molybdenum cofactor in XDH and AOX1, key enzymes involved in the degradation of thiopurines^33^. Previous studies have suggested a role for *MOCOS* gene in thiopurine metabolism with possible impact on clinical outcomes in patients with mutations, however an association between *MOCOS* and TPMT enzyme activity has not been explored. This is the first study to identify a significant role for this gene with variations causing alterations in biochemical enzyme activity. Further work is required to determine how *MOCOS* influences *TPMT* function.

We also detected a nominal association between *GMPS* and drug tolerance (p = 0.0212). *GMPS* is involved in the phosphorylation of 6-TIMP (6-thioinosine monophosphate) to thioguanine nucleotides, which is an important step for thiopurines to exert their cytotoxic effects. Further mechanistic studies will be required to elucidate the molecular mechanisms and clearly define the role of these genes involved in the thiopurine metabolic pathway.

A limitation of this study is that only children who had undergone exome analysis with concurrent TPMT values were selected for the study. While approximately half of our cohort patients had TPMT activity in the intermediate range, only 20% of control samples had TPMT activity in the intermediate category. Exome sequencing was preferentially conducted on children with the most severe disease phenotype, which would have enriched for patients with poor response to first-line treatments. Secondly, the selected population is predominantly Caucasian and a relatively small cohort, thereby limiting the general applicability of the results. Following a systematic search of literature to identify genes implicated in thiopurine toxicity, a panel of 15 genes was prioritised for assessment. It is it is possible that potentially pathogenic variants in other genes may have been missed through this approach. However extension of the list of genes examined would compromise power in the highest priority candidate genes.

Although there is no clear advantage of NGS over the biochemical test in predicting toxicity, our study demonstrates the strength of NGS as a powerful tool in identifying pathogenic variants in patients not detected through standard genotyping. As high throughput sequencing becomes more accessible and affordable, a more objective approach to assessing pharmaco-genomic variation in all genes involved in the drug metabolism pathway may be indicated in selected patients to guide treatment strategies. Replication in larger studies would be required for a comprehensive curation of candidate genes, possibly paving the way for a targeted gene panel as a reliable predictor of toxicity.

## Additional Information

**How to cite this article**: Coelho, T. *et al.* Genes implicated in thiopurine-induced toxicity: Comparing TPMT enzyme activity with clinical phenotype and exome data in a paediatric IBD cohort. *Sci. Rep.* **6**, 34658; doi: 10.1038/srep34658 (2016).

## Supplementary Material

Supplementary Information

## Figures and Tables

**Table 1:** A summary of the key clinical and biochemical (TPMT) features of the cohort.

Clinical Category		Number of patients within each group	Median duration of follow-up in months	Males (%)	Disease			TPMT biochemical activity Number of patients across groups (%)			
							CD (%)	UC (%)	IBDU (%)	Low activity (<10 mU/L)	Intermediate activity (10–67 mU/L)	Normal activity (68–150 mU/L)	High activity (>150 mU/L)
Not Treated with thiopurines		22	60 (10–156)	15 (68)	13 (59)	6 (27)	3 (14)	0 (0)	12 (54)	10 (46)	0
Intolerant		14	42 (9–82)	10(71)	12 (86)	2 (14)	0	0 (0)	8 (57)	6(43)	0
Tolerant	Responders	51	52 (7–124)	24 (47)	31 (60)	13 (26)	7 (14)	0 (0)	22 (43)	28 (55)	1 (2)
	Non-responders	13	63 (13–126)	7 (54)	11 (85)	2 (15)	0	0 (0)	10 (77)	3 (23)	0
	Total	100	54 (7–156)	56 (56)	67 (67)	23 (23)	10 (10)	0 (0)	52 (52)	47 (47)	1 (1)

Of the 100 patients within the cohort, 67 individuals had Crohn’s disease (CD), 23 had ulcerative colitis (UC) and 10 had inflammatory bowel disease unclassified (IBDU). The proportion of males was 56% and the median duration of follow up was 54 months.

**Table 2:** TPMT variants in our cohort.

position in hg19	variant	Coding change	Protein change	Phylop	gerp	MaxEnt	dbSNP	Frequency in 1000 genome	Genotypes in whole cohort (n = 100)	Genotypes in intolerant (n = 14)	Genotypes in tolerant (n = 64)	Mean *TPMT* biochemical value (n=100)
18148069	ns	c.218C > T	p.A73V	0.99	4.98	.	.	.	99,1,0	13;1;0	64;0;0	55
18139272	sp	c.420–4G > A	.	.	.	0.95	.	0.0005	99,1,0	14;0;0	63;1;0	32
**18139228**	**ns**	**c.460G > A**	**p.A154T**	**0.22**	**0.93**	.	**rs1800460**	**0.02**	**91,9,0**	**10;4;0**†	**60;4;0**†	**36.6**
**18130918**	**ns**	**c.719A > G**	**p.Y240C**	**0.99**	**5.13**	.	**rs1142345**	**0.05**	**91,9,0**	**10;4;0**†	**60;4;0**†	**36.6**
18139214	sn	c.474C > T	p.I158I	.	.	.	rs2842934	0.77	6,37,57	1;5;8	3;25;36	67.4

Five *TPMT* variants were identified across the cohort. These included two non-synonymous variants (A154T and Y240C) previously known to impact TMPT function and were found to co-segregate in 9 individuals. A highly pathogenic novel variant was identified in an individual intolerant to thiopurines. The other two included a splicing variant and a common synonymous variant. For all groups, genotypes are listed as homozygous reference allele, heterozygous and homozygous alternative allele. Variants associated with intermediate or low TPMT enzyme activity levels are shown in bold. Novel variants are in grey.

^†^One out of the nine individuals harbouring p.A154T and p. Y240C was not commenced on thiopurine drugs. (Abbreviations: ns- non-synonymous; sn- synonymous; sp- splicing).

**Table 3:** SKAT-O test association analysis across TPMPT and other genes involved in thiopurine toxicity.

Chr	Lbp	Rbp	Gene	Total number of samples	Fraction of individuals who carry rare variants under the MAF thresholds (MAF < 0.05)	Number of all variants defined in the group file	Number of variants defined as rare (MAF < 0.05)*
Biochemical activity (52 with TPMT value <67 and 48 with TPMT value >67)							
18	33767568	33848581	MOCOS	100	0.12000	12	5
6	18130918	18148069	TPMT	100	0.10000	5	2
Tolerance (14 intolerant and 64 tolerant)							
3	155588592	155654236	GMPS	78	0.12821	5	5
6	18130918	18148069	TPMT	78	0.01282	5	1
Responses (51 Responders and 13 non-responders)							
5	55231311	55272085	IL6ST	64	0.10937	8	3
13	95696540	95953517	ABCC4	64	0.31250	22	9

The SKAT-O test was applied to assess the joint effect of common, rare and low frequency variants within the genes implicated in thiopurine toxicity (only significant genes are shown) on TPMT enzyme activity, tolerance and response to the drug.

^*^These variants received different weights in the SKAT-O joint test. Genes are ordered by p-value.

**Table 4:** Deleterious variants occurring within the group of individuals with intolerance to thiopurines.

Gene	Chr	Position on hg19	Variant type	Coding change	Protein change	Novel	Phylop,	1- sift	Polyphen 2	Mutationtaster	Gerp++	dbSNP	Frequency in 1000 genome	Frequency in EVS	TPMT biochemical activity	int	int	int	int	int	int	int	int	Normal	Normal	Normal	Normal	Normal	Normal
															TPMT activity	16	21	47	52	55	18	26	30	80	83	93	113	114	128
															Diagnosis	CD	CD	CD	CD	CD	CD	UC	CD	CD	CD	CD	CD	UC	CD
															Gender	M	M	M	M	F	F	M	F	F	M	M	M	M	M
															IDs	1	3	6	7	8	2	4	5	9	10	11	12	13	14
TPMT	6	18130918	ns	c.719A >G	p.Y240C	.	0.998258	0.94	0.94	0.999973	5.13	rs1142345	0.05	0.041715		1	1	1	1	0	0	0	0	0	0	0	0	0	0
TPMT	6	18148069	ns	c.218C > T	p.A73V	NOVEL	0.999474	0.95	0.898	0.999898	4.98	.	.	.		0	0	0	0	1	0	0	0	0	0	0	0	0	0
MOCOS	18	33779705	ns	c.G359A	p.S120N	.	0.961984	0.98	0.284	0.013289	2.79	rs3744900	0.06	0.047093		0	0	0	0	1	0	0	0	0	0	1	0	0	0
MOCOS	18	33831189	ns	c.C2107A	p.H703N	.	0.998524	0.58	0.706	0.010571	4.98	rs594445	0.25	0.283953		1	0	0	0	0	1	2	0	1	0	1	2	1	0
MOCOS	18	33848581	ns	c.T2600C	p.V867A	.	0.998597	1	0.063	0.006647	5.69	rs1057251	0.08	0.116279		1	0	1	0	0	1	0	1	0	0	0	0	0	0
XDH	2	31572983	ns	c.G2738A	p.R913Q	NOVEL	0.999016	1	1	1	5.38	.	.	.		0	0	0	0	0	1	0	0	0	0	0	0	0	0
XDH	2	31590917	ns	c.A2107G	p.I703V	.	0.998578	1	0.336	0.999913	4.52	rs17011368	0.05	0.034186		0	0	0	0	1	0	0	0	0	0	0	0	0	0
XDH	2	31611143	ns	c.G514A	p.G172R	.	0.998995	0.99	0.004	0.99994	5.35	rs45523133	0.04	0.026047		0	1	0	0	0	0	0	0	0	0	0	0	0	0
XDH	2	31621523	ns	c.A349T	p.T117S	.	0.998756	0.96	0.997	0.999999	5.81	.	.	.		0	0	0	0	0	0	0	0	0	0	0	2	0	0
AOX1	2	2.02E+08	ns	c.A3404G	p.N1135S	.	0.996285	0	0	2.70E-05	5.3	rs55754655	0.11	0.129767		0	0	0	1	0	1	0	0	1	1	0	0	0	1
IL6ST	5	55264153	ns	c.G442C	p.G148R	.	0.995114	0.98	0	2.70E-05	5.6	rs2228044	0.19	0.119535		0	0	1	1	0	0	0	0	0	1	0	0	0	0
NUDT15	13	48619855	ns	c.C415T	p.R139C	.	0.998597	0.92	0.057	0.937718	5.04	rs116855232	0.04	0.002442		0	0	1	0	0	0	0	0	0	0	0	0	0	0
ITPA	20	3193842	ns	c.C94A	p.P32T	.	0.998747	0.87	0.131	0.996149	5.14	rs1127354	0.08	0.067674		0	0	0	0	0	0	0	1	0	0	0	2	0	0
PACSIN2	22	43280404	ns	c.C773A	p.S258Y	.	0.999724	1	0.998	0.999962	5.16	rs200427054	0.0005	0.00314		0	0	0	0	0	0	0	1	0	0	0	0	0	0

Fourteen out of the 100 patients were intolerant to thiopurines. Five of the fourteen individuals had deleterious *TPMT* variants; there was enrichment for deleterious variants within the *MOCOS* gene and the *AOX1* gene in the other 9 individuals. Deleterious variants included: frameshift indels, stopgain/loss, splicing with Maxent score >3 and nonsynonymous variants with a gerp score >2. (ns- non-synonymous; 1 and 2 indicate heterozygous and homozygous genotype respectively).

**Table 5:** Specificity and sensitivity for drug intolerance and tolerance.

		Intolerant	Tolerant	Sensitivity	Specificity
Biochemical test	+	8	32	57.14%	50%
	−	6	32		
Deleterious *TPMT* variants	+	5	4	35.7	93.75%
Non-deleterious variants	−	9	60		

The specificity for predicting toxicity through the biochemical test and through application of TPMT genetic variants was 50% and 93.75% respectively. The sensitivity obtained through both methods was sub-optimal.

**Table 6:** The group of individuals with intolerance to thiopurines.

Patient ID	Diagnosis	Age at Diagnosis (years)	Gender	*TPMT* Gene Deleterious Variants	TPMT Biochemical Activity	TPMT Value (mU/L)	Undergoing treatment with 5-aminosalicylic drugs at time of test	Follow up (months)	Thiopurine Drug	Median Dose (mg/Kg)	Adverse Effects
6	CD	15	M	Yes	Intermediate	21	No	14	AZA	1.5	Leuco-encephalopathy
10	CD	13.5	F	No	Intermediate	18	No	42	AZA	1.5	Neutropenia
22	UC	10.5	M	No	Intermediate	26	Yes	82	AZA	2	Elevated amylase
23	CD	14.5	F	No	Intermediate	30	Yes	63	AZA	2	Persistent Nausea
28	CD	5	M	No	Normal	93	Yes	32	AZA	1	Persistent Nausea
39	CD	10.9	M	Yes	Intermediate	16	No	75	AZA	1.5	Pancytopenia
40	CD	9.5	M	Yes	Intermediate	52	No	75	6-MP	1	Persistent Nausea
41	UC	11.7	M	No	Normal	114	Yes	47	AZA	1	Abnormal ALT
69	CD	16	F	No	Normal	80	No	32	AZA	2.5	Persistent Nausea
73	CD	14	M	No	Normal	128	No	23	AZA	2	Persistent Nausea
74	CD	13.5	F	Yes	Intermediate	55	No	45	AZA	1	Persistent Nausea
88	CD	11	M	No	Normal	83	No	25	AZA	2	Persistent Nausea
92	CD	12	M	Yes	Intermediate	47	No	27	AZA	1.5	Abnormal ALT
95	CD	15.5	M	No	Normal	113	No	9	6-MP	1.5	Pancreatitis

The drugs used were azathioprine (AZA) in 12 patients (dose range 1–2.5 mg/kg/day) and 6-mercaptopurine (6-MP) in 2 patients (dose range 1–1.5 mg/kg/day). The choice of drug was based on clinician preference.
