# Supplementary material for: Genes implicated in thiopurine-induced toxicity: Comparing TPMT enzyme activity with clinical phenotype and exome data in a paediatric IBD cohort
Source: Sci Rep. 2016 Oct 5;6:34658. doi: 10.1038/srep34658 (PMC5050412; doi:10.1038/srep34658)
Supplement: Supplementary Information [file srep34658-s1.pdf]

## Title:

**Genes implicated in thiopurine-induced toxicity: Comparing TPMT enzyme activity with clinical phenotype and exome data in a paediatric IBD cohort**

**Correspondence to:**

*Dr Tracy Coelho

Human Genetics & Genomic Medicine, Duthie Building (Mp 808)

University Hospital Southampton Foundation Trust, Southampton

SO16 6YD, UK

**Telephone:** +44 (0) 23 81208614 **E-Mail:** T.F.Coelho@soton.ac.uk

**Authors:**

[$]Tracy Coelho[1, 2]

[$]Gaia Andreoletti [1]

([$]Joint first authors)

James J. Ashton [2]

Akshay Batra [2]

Nadeem Afzal[2]

Yifang Gao[3]

Anthony P. Williams[3]

Robert M. Beattie [2]

Sarah Ennis[1]

**Affiliations of all authors:**

[1] Human Genetics and Genomic medicine, University of Southampton, Southampton, UK

[2] Department of Paediatric Gastroenterology, University Hospital Southampton

[3] Cancer Sciences Division, Faculty of Medicine, University Hospital Southampton

**Supplementary tables**

**Table S1: Genes implicated in thiopurine metabolism and toxicity**

| | **HUGO Gene** | **Symbol** | **Chromosome** | **Function** | **Percentage coverage Agilent v5** | **Percentage coverage Agilent v4** | **References** |
|---|---|---|---|---|---|---|---|
| 1 | Aldehyde oxidase 1 | *AOX1* | 2 | Catabolism of 6-MP to 6-thiouracil (6-TU) | 100 | 88.87 | [1-3] |
| 2 | ATP-binding cassette, sub-family C (CFTR/MRP), member 4 | *ABCC4* | 13 | Efflux pump transporting a wide variety of endogenous and xenobiotic organic anionic compounds including 6-MP and 6-TGN out of the cell | 95.61 | 72.00 | [4] |
| 3 | Follistatin-Like 5 | *FSTL5* | 4 | Involved in calcium ion binding and interacting with metalloproteases at the extracellular matrix level | 91.40 | 57.40 | [5] |
| 4 | Guanine monphosphate synthase | *GMPS* | 3 | Converts 6-TIMP to 6-thioguanines (6-TGN) | 100 | 96.78 | [6] |
| 5 | Glutathione-s-transferase | *GSTM1* | 1 | Detoxification enzymes catalysing the conjugation of electrophilic substrates to glutathione | 100 | 42 | [7-10] |
| 6 | Hypoxanthine phosphoribosyl transferase 1 | *HPRT1* | X | Conversion of 6-MP to 6-TIMP | 100 | 76.81 | [6, 11-15] |
| 7 | Inosine-5-Monophosphate Dehydrogenase 1 | *IMPDH1* | 7 | Converts 6-TIMP to 6-thioguanines (6-TGN) | 99.39 | 77.41 | [6, 16] |
| 8 | Inosine-5-Monophosphate Dehydrogenase 2 | *IMPDH2* | X | Converts 6-TIMP to 6-thioguanines (6-TGN) | 100 | 100 | [6, 16] |
| 9 | Inosine triphosphatase (nucleoside triphosphate pyrophosphatase) | *ITPA* | 20 | De-phosphorylation of 6-thioinosine triphosphate (6-TITP) to 6-thioinosine monophosphate (6-TIMP) in the thiopurine metabolic pathway | 89.65 | 76.99 | [4, 6, 7, 11, 17-31] |
| 10 | Interleukin 6 signal transducer | *IL6ST* | 5 | Signal transducer protein shared by several cytokines including IL-6 | 96.55 | 32.14 | [5] |
| 11 | Methylenetetrahydrofolate reductase | *MTHFR* | 1 | Intracellular folate metabolism and purine-pyrimidine synthesis | 95.3 | 65.70 | [32] |
| 12 | Molybdenum cofactor sulfurase | *MOCOS* | 18 | Sulfuration of molybdenum co-factor in the enzymes XDH and AOX1; essential for their enzymatic activities | 100 | 100 | [2, 3] |
| 13 | Nudix (nucleoside diphosphate linked moiety X)-type motif 15 | *NUDT15* | 13 | Involved in DNA repair | 95.99 | 43.12 | [33] |

| | | | | | | | |
|---|---|---|---|---|---|---|---|
| 14 | Protein kinase C and casein kinase substrate in neurons 2 | *PACSIN2* | 22 | Family of proteins, involved in various biological processes including endocytosis, cell-cycle control and autophagy | 89.01 | 49.78 | [34] |
| 15 | Xanthine dehydrogenase (Synonyms- XO) | *XDH* | 2 | Catabolism of 6-MP to 6-thiouracil (6-TU) | 100 | 75.94 | [1-3, 12, 35, 36] |

Fifteen genes implicated in thiopurine metabolism and toxicity identified through a systematic search shown in the table (tabulated in an alphabetical order). The *HLA-DQA1-HLA-DRB1* locus although implicated in thiopurine toxicity, was not included in our analysis due to inherent difficulties in analysis of this highly polymorphic region through whole-exome sequencing data.

**Table S2: Specificity and sensitivity for predicting normality of the biochemical test**

| | | TPMT value < 67 | TPMT value > 67 | Sensitivity | Specificity |
|---|---|---|---|---|---|
| Deleterious *TPMT* variants | + | 10 | 0 | 19.23% | 100% |
| Non-deleterious *TPMT* variants | - | 42 | 48 | | |
| Deleterious *MOCOS* variants | + | 28 | 30 | 60.86% | 37.50% |
| Non-deleterious *MOCOS* variants | - | 24 | 18 | | |

In this table, the sensitivity and specificity values for predicting the normality of the TPMT enzyme activity (with 67 mU/L as the cut-off between low or intermediate and normal) through application of the TPMT variants alone or in combination with the MOCOS variants. Inclusion of the MOCOS variants to the TPMT variation improves the sensitivity for the biochemical activity level of TPMT, but a corresponding loss of specificity is observed when compared with the TPMT variation alone.

**Table S3: Specificity and sensitivity for Responders and non-responders**

| | | Responders | Non-Responders | Sensitivity | Specificity |
|---|---|---|---|---|---|
| Deleterious *IL6ST* variants | + | 14 | 2 | 27.45% | 84.62% |
| Non-deleterious *IL6ST* variants | - | 37 | 11 | | |
| Deleterious *ABCC4* variants | + | 4 | 6 | 7.84% | 53.85% |
| Non-deleterious *ABCC4* variants | - | 47 | 7 | | |

Table S3 demonstrates the sensitivity and specificity of application of IL6ST and ABCC4 variations with clinical response to thiopurines as the gold standard.

## Table S4: Clinical Information on the 100 patients recruited to the study

| Patients | Gender | Diagnosis | TPMT mU/L | Any blood transfusions before the test (<3 months) | On 5-ASA drugs at or before the time of test | On Azathioprine/6-Mercaptopurine | Dose (mg/kg) | Abnormal counts requiring withdrawal of the drug | Abnormal LFTS after starting the drug | Pancreatitis | Other complications | Drug withdrawn | Possible Responder | Possible Nonresponder or Poor responder | Therapy intolerant |
|---|---|---|---|---|---|---|---|---|---|---|---|---|---|---|---|
| 1 | M | CD | 34 | X | X | Azathioprine | 2.75 | X | X | X | X | X | X | ✓ | X |
| 2 | F | CD | 31 | X | X | Azathioprine | 2 | X | X | X | X | X | ✓ | X | X |
| 3 | M | CD | 32 | X | X | Azathioprine | 2.3 | X | X | X | X | X | ✓ | X | X |
| 4 | M | CD | 55 | X | X | Azathioprine | 2.2 | X | X | X | X | X | ✓ | X | X |
| 5 | M | CD | 32 | X | X | Azathioprine | 2 | X | X | X | X | X | X | ✓ | X |
| 6 | M | CD | 21 | X | X | Azathioprine | 1.5 | X | X | X | Leucoencephalopathy | X | X | X | ✓ |
| 7 | M | CD | 38 | X | X | None | Not Applicable | Not Applicable | Not Applicable | Not Applicable | Not Applicable | Not Applicable | Not Applicable | Not Applicable | Not Applicable |
| 8 | M | UC | 33 | X | ✓ | Azathioprine | 2.7 | X | X | X | X | X | ✓ | X | X |
| 9 | M | CD | 25 | X | X | Azathioprine | 2 | X | X | X | X | X | ✓ | X | X |
| 10 | F | CD | 18 | X | X | Azathioprine | 2 | ✓ | X | X | Neutropenia | ✓ | X | X | ✓ |
| 11 | F | CD | 49 | X | ✓ | Azathioprine | 2.5 | X | X | X | X | X | X | ✓ | X |
| 12 | M | CD | 29 | X | ✓ | Azathioprine | 2.5 | X | X | X | X | ✓ | X | ✓ | X |
| 13 | M | CD | 29 | X | ✓ | None | Not Applicable | Not Applicable | Not Applicable | Not Applicable | Not Applicable | Not Applicable | Not Applicable | Not Applicable | Not Applicable |
| 14 | F | CD | 25 | X | X | Azathioprine | 2 | X | X | X | X | X | X | ✓ | X |
| 15 | M | UC | 101 | X | ✓ | Azathioprine | 2.5 | X | X | X | X | X | ✓ | X | X |
| 16 | M | UC | 77 | X | ✓ | None | Not Applicable | Not Applicable | Not Applicable | Not Applicable | Not Applicable | Not Applicable | Not Applicable | Not Applicable | Not Applicable |
| 17 | F | CD | 85 | X | X | Azathioprine | 2 | X | X | X | X | X | ✓ | X | X |
| 18 | M | CD | 32 | X | X | Azathioprine | 2.5 | X | X | X | X | X | X | ✓ | X |
| 19 | M | CD | 32 | X | X | Azathioprine | 2 | X | X | X | X | X | X | ✓ | X |
| 20 | M | CD | 29 | X | X | Azathioprine | 2 | X | X | X | X | X | ✓ | X | X |
| 21 | F | CD | 32 | X | ✓ | Azathioprine | 2.25 | X | X | X | X | X | ✓ | X | X |
| 22 | M | UC | 26 | X | ✓ | Azathioprine | 2 | X | X | X | Elevated amylase | ✓ | X | X | ✓ |
| 23 | F | CD | 30 | X | ✓ | Azathioprine | 2 | X | X | X | Severe nausea | ✓ | X | X | ✓ |
| 24 | M | CD | 138 | X | ✓ | Azathioprine | 2 | X | X | X | X | ✓ | ✓ | X | X |
| 25 | M | UC | 37 | ✓ | X | None | Not Applicable | Not Applicable | Not Applicable | Not Applicable | Not Applicable | Not Applicable | Not Applicable | Not Applicable | Not Applicable |
| 26 | M | CD | 127 | X | X | None | Not Applicable | Not Applicable | Not Applicable | Not Applicable | Not Applicable | Not Applicable | Not Applicable | Not Applicable | Not Applicable |
| 27 | M | UC | 29 | X | X | Azathioprine | 2 | X | X | X | X | X | ✓ | X | X |
| 28 | M | CD | 93 | X | ✓ | Azathioprine | 1 | X | X | X | Severe nausea | ✓ | X | X | ✓ |
| 29 | M | UC | 17 | X | X | Azathioprine | 2.5 | X | X | X | X | X | ✓ | X | X |
| 30 | M | CD | 37 | X | ✓ | Azathioprine | 1.5 | X | X | X | X | X | ✓ | X | X |
| 31 | M | CD | 34 | X | X | Azathioprine | 1.5 | X | X | X | X | X | ✓ | X | X |
| 32 | F | UC | 34 | X | ✓ | None | Not Applicable | Not Applicable | Not Applicable | Not Applicable | Not Applicable | Not Applicable | Not Applicable | Not Applicable | Not Applicable |
| 33 | M | CD | 18 | X | ✓ | Azathioprine | 2 | X | X | X | X | X | ✓ | X | X |
| 34 | F | UC | 29 | X | ✓ | 6-Mercaptopurine | 1 | X | ✓ | X | X | X | X | ✓ | X |
| 35 | F | IBDU | 105 | X | X | 6-Mercaptopurine | 1 | X | X | X | X | X | ✓ | X | X |
| 36 | F | UC | 92 | X | ✓ | 6-Mercaptopurine | 2 | X | X | X | X | X | ✓ | X | X |
| 37 | F | CD | 41 | X | X | Azathioprine | 2 | X | X | X | X | X | ✓ | X | X |
| 38 | F | UC | 30 | X | X | 6-Mercaptopurine | 2 | X | X | X | X | X | ✓ | X | X |
| 39 | M | CD | 16 | X | X | Azathioprine | 1.5 | ✓ | X | X | Pancytopenia | ✓ | X | X | ✓ |
| 40 | M | CD | 52 | X | ✓ | 6-MP | 1 | X | X | X | Severe nausea | ✓ | X | X | ✓ |
| 41 | M | UC | 114 | X | ✓ | Azathioprine | 1 | X | ✓ | X | Abnormal LFTs | ✓ | X | X | X |
| 42 | M | CD | 93 | X | ✓ | None | Not Applicable | Not Applicable | Not Applicable | Not Applicable | Not Applicable | Not Applicable | Not Applicable | Not Applicable | Not Applicable |
| 43 | F | UC | 50 | X | ✓ | Azathioprine | 1.5 | X | X | X | X | X | ✓ | X | X |
| 44 | F | CD | 36 | X | X | 6-Mercaptopurine | 1 | X | X | X | X | X | ✓ | X | X |
| 45 | M | UC | 25 | X | X | None | Not Applicable | Not Applicable | Not Applicable | Not Applicable | Not Applicable | Not Applicable | Not Applicable | Not Applicable | Not Applicable |
| 46 | F | UC | 31 | X | X | 6-Mercaptopurine | 1.5 | X | X | X | X | X | X | ✓ | X |
| 47 | F | CD | 96 | X | X | Azathioprine | 1.5 | X | X | X | X | X | ✓ | X | X |
| 48 | F | UC | 105 | X | ✓ | Azathioprine | 2 | X | X | X | X | X | ✓ | X | X |
| 49 | F | CD | 27 | X | X | None | Not Applicable | Not Applicable | Not Applicable | Not Applicable | Not Applicable | Not Applicable | Not Applicable | Not Applicable | Not Applicable |

| | | | | | | | | | | | | | | | |
|---|---|---|---|---|---|---|---|---|---|---|---|---|---|---|---|
| **50** | F | IBDU | 91 | X | ✓ | None | Not Applicable | Not Applicable | Not Applicable | Not Applicable | Not Applicable | Not Applicable | Not Applicable | Not Applicable | Not Applicable |
| **51** | F | IBDU | 82 | X | ✓ | 6-Mercaptopurine | 1.5 | X | X | X | X | X | ✓ | X | X |
| **52** | M | CD | 14 | X | X | None | Not Applicable | Not Applicable | Not Applicable | Not Applicable | Not Applicable | Not Applicable | Not Applicable | Not Applicable | Not Applicable |
| **53** | M | CD | 85 | X | X | 6-Mercaptopurine | 1 | X | X | X | X | X | ✓ | X | X |
| **54** | F | UC | 152 | X | ✓ | Azathioprine | 2.5 | X | X | X | X | X | ✓ | X | X |
| **55** | F | CD | 86 | X | X | None | Not Applicable | Not Applicable | Not Applicable | Not Applicable | Not Applicable | Not Applicable | Not Applicable | Not Applicable | Not Applicable |
| **56** | F | CD | 101 | X | X | 6-Mercaptopurine | 1 | X | X | X | X | X | ✓ | X | X |
| **57** | M | UC | 26 | X | X | None | Not Applicable | Not Applicable | Not Applicable | Not Applicable | Not Applicable | Not Applicable | Not Applicable | Not Applicable | Not Applicable |
| **58** | F | CD | 77 | X | X | Azathioprine | 2.5 | X | X | X | X | X | X | ✓ | X |
| **59** | F | CD | 119 | X | X | 6-Mercaptopurine | 1 | X | X | X | X | X | ✓ | X | X |
| **60** | F | UC | 38 | X | ✓ | None | Not Applicable | Not Applicable | Not Applicable | Not Applicable | Not Applicable | Not Applicable | Not Applicable | Not Applicable | Not Applicable |
| **61** | F | IBDU | 92 | X | X | Azathioprine | 2.5 | X | X | X | X | X | ✓ | X | X |
| **62** | F | IBDU | 95 | X | X | None | Not Applicable | Not Applicable | Not Applicable | Not Applicable | Not Applicable | Not Applicable | Not Applicable | Not Applicable | Not Applicable |
| **63** | M | CD | 73 | X | X | Azathioprine | 2 | X | X | X | X | X | ✓ | X | X |
| **64** | F | CD | 66 | X | X | 6-Mercaptopurine | 1 | X | X | X | X | X | ✓ | X | X |
| **65** | M | CD | 30 | X | X | Azathioprine | 2 | X | X | X | X | X | ✓ | X | X |
| **66** | M | CD | 38 | X | X | None | Not Applicable | Not Applicable | Not Applicable | Not Applicable | Not Applicable | Not Applicable | Not Applicable | Not Applicable | Not Applicable |
| **67** | F | CD | 66 | X | X | Azathioprine | 2 | X | X | X | X | X | ✓ | X | X |
| **68** | F | CD | 124 | X | ✓ | Azathioprine | 2.5 | X | ✓ | X | Autoimmune sclerosing cholangitis | ✓ | ✓ | X | X |
| **69** | F | CD | 80 | X | X | 6-Mercaptopurine | 2.5 | X | X | X | Severe nausea | X | X | X | ✓ |
| **70** | M | CD | 96 | X | X | Azathioprine | 2 | X | X | X | X | ✓ | X | ✓ | X |
| **71** | M | CD | 90 | X | X | 6-Mercaptopurine | 1 | X | ✓ | X | X | X | ✓ | X | X |
| **72** | F | IBDU | 35 | X | X | 6-Mercaptopurine | 1 | X | X | X | X | X | ✓ | X | X |
| **73** | M | CD | 128 | X | X | Azathioprine | 2 | X | X | X | Severe nausea | ✓ | X | X | ✓ |
| **74** | F | CD | 55 | X | X | Azathioprine | 1 | X | X | X | Severe nausea | ✓ | X | X | ✓ |
| **75** | F | CD | 97 | X | X | Azathioprine | 2 | X | ✓ | X | X | X | ✓ | X | X |
| **76** | M | CD | 70 | X | X | None | Not Applicable | Not Applicable | Not Applicable | Not Applicable | Not Applicable | Not Applicable | Not Applicable | Not Applicable | Not Applicable |
| **77** | M | IBDU | 99 | X | X | 6-Mercaptopurine | 1 | X | X | X | X | X | ✓ | X | X |
| **78** | F | CD | 82 | X | X | Azathioprine | 2 | X | X | X | X | X | ✓ | X | X |
| **79** | F | CD | 87 | X | X | 6-Mercaptopurine | 1 | X | X | X | X | X | ✓ | X | X |
| **80** | M | UC | 53 | X | X | Azathioprine | 1 | X | X | X | X | X | ✓ | X | X |
| **81** | M | CD | 110 | X | ✓ | Azathioprine | 2.5 | X | X | X | X | X | ✓ | X | X |
| **82** | F | IBDU | 100 | X | X | Azathioprine | 2 | X | X | X | X | X | ✓ | X | X |
| **83** | F | CD | 99 | X | X | 6-Mercaptopurine | 1 | X | X | X | X | X | ✓ | X | X |
| **84** | M | CD | 28 | X | X | Azathioprine | 2 | X | X | X | X | X | ✓ | X | X |
| **85** | M | CD | 85 | X | X | Azathioprine | 2 | X | X | X | X | X | ✓ | X | X |
| **86** | M | CD | 101 | X | X | None | Not Applicable | Not Applicable | Not Applicable | Not Applicable | Not Applicable | Not Applicable | Not Applicable | Not Applicable | Not Applicable |
| **87** | M | CD | 100 | X | X | Azathioprine | 2 | X | X | X | X | X | ✓ | X | X |
| **88** | M | CD | 83 | X | X | Azathioprine | 2 | X | X | X | Severe nausea | ✓ | X | X | ✓ |
| **89** | M | UC | 96 | X | X | Azathioprine | 2 | X | X | X | ▯ | X | ✓ | X | X |
| **90** | M | CD | 145 | X | X | None | Not Applicable | Not Applicable | Not Applicable | Not Applicable | Not Applicable | Not Applicable | Not Applicable | Not Applicable | Not Applicable |
| **91** | M | CD | 40 | X | X | Azathioprine | 1.5 | X | X | X | X | X | X | ✓ | X |
| **92** | M | CD | 47 | X | X | Azathioprine | 1.5 | X | ✓ | X | Abnormal LFTs | ✓ | X | X | ✓ |
| **93** | M | CD | 35 | X | X | None | Not Applicable | Not Applicable | Not Applicable | Not Applicable | Not Applicable | Not Applicable | Not Applicable | Not Applicable | Not Applicable |
| **94** | F | CD | 102 | X | X | Azathioprine | 2 | X | X | X | X | X | X | ✓ | X |
| **95** | M | CD | 113 | X | X | 6-Mercaptopurine | 1.5 | X | X | ✓ | Pancreatitis | ✓ | X | X | ✓ |
| **96** | F | UC | 89 | X | X | 6-Mercaptopurine | 1 | X | X | X | X | X | ✓ | X | X |
| **97** | F | IBDU | 56 | X | ✓ | 6-Mercaptopurine | 1 | X | X | X | X | X | ✓ | X | X |
| **98** | M | CD | 86 | X | X | None | Not Applicable | Not Applicable | Not Applicable | Not Applicable | Not Applicable | Not Applicable | Not Applicable | Not Applicable | Not Applicable |
| **99** | F | CD | 80 | X | X | None | Not Applicable | Not Applicable | Not Applicable | Not Applicable | Not Applicable | Not Applicable | Not Applicable | Not Applicable | Not Applicable |
| **100** | M | UC | 99 | X | X | Azathioprine | 2 | X | X | X | ▯ | X | ▯ | X | X |

Table S4 shows the clinical details on all 100 patients within the cohort

**References:**


1. Choughule, K.V., et al., *In vitro oxidative metabolism of 6-mercaptopurine in human liver: insights into the role of the molybdoflavoenzymes aldehyde oxidase, xanthine oxidase, and xanthine dehydrogenase.* Drug Metab Dispos, 2014. **42**(8): p. 1334-40.
2. Kurzawski, M., et al., *Polymorphism of genes involved in purine metabolism (XDH, AOX1, MOCOS) in kidney transplant recipients receiving azathioprine.* Ther Drug Monit, 2012. **34**(3): p. 266-74.
3. Smith, M.A., et al., *Novel pharmacogenetic markers for treatment outcome in azathioprine-treated inflammatory bowel disease.* Aliment Pharmacol Ther, 2009. **30**(4): p. 375-84.
4. Ban, H., et al., *The multidrug-resistance protein 4 polymorphism is a new factor accounting for thiopurine sensitivity in Japanese patients with inflammatory bowel disease.* J Gastroenterol, 2010. **45**(10): p. 1014-21.
5. Zabala, W., et al., *New genetic associations in thiopurine-related bone marrow toxicity among inflammatory bowel disease patients.* Pharmacogenomics, 2013. **14**(6): p. 631-40.
6. Kudo, M., et al., *Genetic variations in the HGPRT, ITPA, IMPDH1, IMPDH2, and GMPS genes in Japanese individuals.* Drug Metab Pharmacokinet, 2009. **24**(6): p. 557-64.
7. Stocco, G., et al., *Deletion of glutathione-s-transferase m1 reduces azathioprine metabolite concentrations in young patients with inflammatory bowel disease.* J Clin Gastroenterol, 2014. **48**(1): p. 43-51.
8. Mazor, Y., et al., *Risk factors for serious adverse effects of thiopurines in patients with Crohn's disease.* Curr Drug Saf, 2013. **8**(3): p. 181-5.
9. Stocco, G., et al., *Glutathione-S-transferase genotypes and the adverse effects of azathioprine in young patients with inflammatory bowel disease.* Inflamm Bowel Dis, 2007. **13**(1): p. 57-64.
10. Zhang, W., O. Moden, and B. Mannervik, *Differences among allelic variants of human glutathione transferase A2-2 in the activation of azathioprine.* Chem Biol Interact, 2010. **186**(2): p. 110-7.

11. Palmieri, O., et al., *Sequential evaluation of thiopurine methyltransferase, inosine triphosphate pyrophosphatase, and HPRT1 genes polymorphisms to explain thiopurines' toxicity and efficacy.* Aliment Pharmacol Ther, 2007. **26**(5): p. 737-45.
12. Seinen, M.L., et al., *The effect of allopurinol and low-dose thiopurine combination therapy on the activity of three pivotal thiopurine metabolizing enzymes: results from a prospective pharmacological study.* J Crohns Colitis, 2013. **7**(10): p. 812-9.
13. Devyatko, E., et al., *Activation of the purine salvage pathway in mononuclear cells of cardiac recipients treated with mycophenolate mofetil.* Transplantation, 2006. **82**(1): p. 113-8.
14. Ding, L., et al., *Hypoxanthine guanine phosphoribosyltransferase activity is related to 6-thioguanine nucleotide concentrations and thiopurine-induced leukopenia in the treatment of inflammatory bowel disease.* Inflamm Bowel Dis, 2012. **18**(1): p. 63-73.
15. van Asseldonk, D.P., et al., *Limited intra-individual variability in hypoxanthine-Guanine phosphoribosyl transferase, thiopurine S-methyl transferase, and xanthine oxidase activity in inflammatory bowel disease patients during 6-thioguanine therapy.* Nucleosides Nucleotides Nucleic Acids, 2010. **29**(4-6): p. 284-90.
16. Haglund, S., et al., *The role of inosine-5'-monophosphate dehydrogenase in thiopurine metabolism in patients with inflammatory bowel disease.* Ther Drug Monit, 2011. **33**(2): p. 200-8.
17. Farfan, M.J., et al., *Prevalence of TPMT and ITPA gene polymorphisms and effect on mercaptopurine dosage in Chilean children with acute lymphoblastic leukemia.* BMC Cancer, 2014. **14**: p. 299.
18. Bierau, J., et al., *Determination of ITPase activity in erythrocyte lysates obtained for determination of TPMT activity.* Nucleosides Nucleotides Nucleic Acids, 2006. **25**(9-11): p. 1129-32.
19. Zelinkova, Z., et al., *Inosine triphosphate pyrophosphatase and thiopurine s-methyltransferase genotypes relationship to azathioprine-induced myelosuppression.* Clin Gastroenterol Hepatol, 2006. **4**(1): p. 44-9.

20. von Ahsen, N., et al., *Association of inosine triphosphatase 94C>A and thiopurine S-methyltransferase deficiency with adverse events and study drop-outs under azathioprine therapy in a prospective Crohn disease study.* Clin Chem, 2005. **51**(12): p. 2282-8.

21. Marinaki, A.M., et al., *Adverse drug reactions to azathioprine therapy are associated with polymorphism in the gene encoding inosine triphosphate pyrophosphatase (ITPase).* Pharmacogenetics, 2004. **14**(3): p. 181-7.

22. Buster, E.H., et al., *Thiopurine-methyltransferase and inosine triphosphate pyrophosphatase polymorphism in a liver transplant recipient developing nodular regenerative hyperplasia on low-dose azathioprine.* Eur J Gastroenterol Hepatol, 2008. **20**(1): p. 68-72.

23. Stocco, G., et al., *Genetic polymorphism of inosine triphosphate pyrophosphatase is a determinant of mercaptopurine metabolism and toxicity during treatment for acute lymphoblastic leukemia.* Clin Pharmacol Ther, 2009. **85**(2): p. 164-72.

24. Uchiyama, K., et al., *Thiopurine S-methyltransferase and inosine triphosphate pyrophosphohydrolase genes in Japanese patients with inflammatory bowel disease in whom adverse drug reactions were induced by azathioprine/6-mercaptopurine treatment.* J Gastroenterol, 2009. **44**(3): p. 197-203.

25. Cheon, J.H., et al., *Allele frequency of thiopurine methyltransferase and inosine triphosphate pyrophosphatase gene polymorphisms in Korean patients with inflammatory bowel diseases.* Hepatogastroenterology, 2009. **56**(90): p. 421-3.

26. Jung, Y.S., et al., *Correlation of genotypes for thiopurine methyltransferase and inosine triphosphate pyrophosphatase with long-term clinical outcomes in Korean patients with inflammatory bowel diseases during treatment with thiopurine drugs.* J Hum Genet, 2010. **55**(2): p. 121-3.

27. Xiong, H., et al., *Association between inosine triphosphate pyrophosphohydrolase deficiency and azathioprine-related adverse drug reactions in the Chinese kidney transplant recipients.* Fundam Clin Pharmacol, 2010. **24**(3): p. 393-400.

28. Zabala-Fernandez, W., et al., *A pharmacogenetics study of TPMT and ITPA genes detects a relationship with side effects and clinical response in patients with inflammatory bowel disease receiving Azathioprine.* J Gastrointestin Liver Dis, 2011. **20**(3): p. 247-53.

29. Wan Rosalina, W.R., et al., *Polymorphism of ITPA 94C>A and risk of adverse effects among patients with acute lymphoblastic leukaemia treated with 6-mercaptopurine.* J Clin Pharm Ther, 2012. **37**(2): p. 237-41.

30. Dorababu, P., et al., *Epistatic interactions between thiopurine methyltransferase (TPMT) and inosine triphosphate pyrophosphatase (ITPA) variations determine 6-mercaptopurine toxicity in Indian children with acute lymphoblastic leukemia.* Eur J Clin Pharmacol, 2012. **68**(4): p. 379-87.

31. Melaouhia, S., et al., *Allele frequency of inosine triphosphate pyrophosphatase (ITPA) and thiopurine-S-methyl transferase (TPMT) genes in the Tunisian population.* Clin Res Hepatol Gastroenterol, 2012. **36**(2): p. 178-84.

32. Karas-Kuzelicki, N., et al., *Heterozygosity at the TPMT gene locus, augmented by mutated MTHFR gene, predisposes to 6-MP related toxicities in childhood ALL patients.* Leukemia, 2009. **23**(5): p. 971-4.

33. Yang, S.K., et al., *A common missense variant in NUDT15 confers susceptibility to thiopurine-induced leukopenia.* Nat Genet, 2014. **46**(9): p. 1017-20.

34. Stocco, G., et al., *PACSIN2 polymorphism influences TPMT activity and mercaptopurine-related gastrointestinal toxicity.* Hum Mol Genet, 2012. **21**(21): p. 4793-804.

35. Kalra, S., et al., *Preferential inhibition of xanthine oxidase by 2-amino-6-hydroxy-8-mercaptopurine and 2-amino-6-purine thiol.* BMC Biochem, 2007. **8**: p. 8.

36. Wong, D.R., et al., *The role of xanthine oxidase in thiopurine metabolism: a case report.* Ther Drug Monit, 2007. **29**(6): p. 845-8.
